# A Bluetooth-Based Smartphone App for Detecting Peer Proximity: Protocol for Evaluating Functionality and Validity

**DOI:** 10.2196/50241

**Published:** 2024-04-05

**Authors:** Nancy P Barnett, Alexander W Sokolovsky, Matthew K Meisel, Shannon R Forkus, Kristina M Jackson

**Affiliations:** 1 Department of Behavioral and Social Sciences Brown University Providence, RI United States; 2 Department of Psychiatry and Behavioral Sciences Medical University of South Carolina Charleston, SC United States

**Keywords:** Bluetooth technology, passive sensing, proximity detection, ecological momentary assessment, social influence, alcohol use, mobile phone

## Abstract

**Background:**

While ecological momentary assessment (EMA) is commonly used to study social contexts and social influence in the real world, EMA almost exclusively relies on participant self-report of present circumstances, including the proximity to influential peers. There is the potential for developing a proximity sensing approach that uses small Bluetooth beacons and smartphone-based detection and data collection to collect information about interactions between individuals passively in real time.

**Objective:**

This paper aims to describe the methods for evaluating the functionality and validity of a Bluetooth-based beacon and a smartphone app to identify when ≥2 individuals are physically proximal.

**Methods:**

We will recruit 20 participants aged 18 to 29 years with Android smartphones to complete a 3-week study during which beacon detection and self-report data will be collected using a smartphone app (MEI Research). Using an interviewer-administered social network interview, participants will identify up to 3 peers of the same age who are influential on health behavior (alcohol use in this study). These peers will be asked to carry a Bluetooth beacon (Kontakt asset tag) for the duration of the study; each beacon has a unique ID that, when detected, will be recorded by the app on the participant’s phone. Participants will be prompted to respond to EMA surveys (signal-contingent reports) when a peer beacon encounter meets our criteria and randomly 3 times daily (random reports) and every morning (morning reports) to collect information about the presence of peers. In all reports, the individualized list of peers will be presented to participants, followed by questions about peer and participant behavior, including alcohol use. Data from multiple app data sets, including beacon encounter specifications, notification, and app logs, participant EMA self-reports and postparticipation interviews, and peer surveys, will be used to evaluate project goals. We will examine the functionality of the technology, including the stability of the app (eg, app crashes and issues opening the app), beacon-to-app detection (ie, does the app detect proximal beacons?), and beacon encounter notification when encounter criteria are met. The validity of the technology will be defined as the concordance between passive detection of peers via beacon-to-app communication and the participant’s EMA report of peer presence. Disagreement between the beacon and self-report data (ie, false negatives and false positives) will be investigated in multiple ways (ie, to determine if the reason was technology-related or participant compliance-related) using encounter data and information collected from participants and peers.

**Results:**

Participant recruitment began in February 2023, and enrollment was completed in December 2023. Results will be reported in 2025.

**Conclusions:**

This Bluetooth-based technology has important applications and clinical implications for various health behaviors, including the potential for just-in-time adaptive interventions that target high-risk behavior in real time.

**International Registered Report Identifier (IRRID):**

DERR1-10.2196/50241

## Introduction

### Behavioral Influence in Social Contexts

Social context, which refers to immediate temporal, situational, and intrapersonal factors, is important for many health behaviors [1]. Understanding how context influences behavior is an essential first step toward the development of preventive interventions to reduce risk, as it provides essential information on why, with whom, where, and when a person engages in a particular behavior. For alcohol use, the health behavior we will focus on, the presence of peers is a highly influential contextual factor for all ages [2-6]. Recent studies have examined real-time information gathered about individuals and their environment [7-9] via purposeful, self-initiated reports or prompted reports [eg, ecological momentary assessment (EMA)]. Compared with retrospective recall, which can lead to error, EMA methods probe for participant reports in real time, leading to more accurate perceptions of behavior and allowing for the assessment of changes in the social context across a day [10]. However, while EMA as a method can help advance our understanding of the social context, it requires individuals to be both aware of and able to report peer presence or influence. Thus, there is value in research that relies less on self-report and more on passive assessment of the social context.

### Use of Technology to Examine Social Contexts

There has been a rapid rise in the passive ambulatory assessment of behavior [11,12] using various technologies, including wearables for physical activity or heart rate [13,14] and alcohol biosensors [15,16]. In addition to wearable sensors, smartphones passively collect data from their built-in sensors in real time [17], including information on location and movement [18-20] and social interactions [20,21]. A smartphone-based technology with potential applications for understanding social contexts is Bluetooth, which is a ubiquitous connectivity protocol embedded in mobile phones and other wearable devices. Designed to underlie communication between digital devices, the unique characteristics of Bluetooth have enabled the development of software that can identify nearby Bluetooth beacons (eg, Apple AirTags or other transmitters, including smartphones themselves), allowing smartphone apps to assess the duration and frequency of interpersonal interactions [22-24].

Despite its promise, little research has applied Bluetooth technology to proximity sensing to study the social contexts of health behavior change. An epidemiological study used smartphone-based Bluetooth sensors to predict behavior change associated with disease spread [25], and another used Bluetooth-based proximity sensing to assess the relationships among sociability, sleep, and mood [26]. A recent study on alcohol use incorporated Bluetooth sensing to examine the social context of young adult drinking, in which the Bluetooth technology captured features such as the number of proximal devices and signal strengths [27]. Recently, researchers have evaluated the performance of Bluetooth-integrated methods to understand disease spread during the COVID-19 pandemic [28-30]. Our goal is to develop and evaluate the stability of a smartphone app that leverages a Bluetooth-based wearable sensing protocol to study the real-world social context of alcohol use but could be applicable to other behaviors in which social contexts act as key determinants.

### Study Objective

The objective of this research is to develop technology that will allow for the passive detection of contact between individuals, and specifically between participants and their close friends. In this paper, we describe a smartphone app, Bluetooth-based beacons, and our planned procedures for evaluating the *functionality and validity* of the developed technology. In a companion paper [31], we describe our approach to evaluating participant responses to using the technology in a study on the social context of alcohol (ie, feasibility and acceptability). *Functionality* will be determined by evaluating the stability of the app (ie, low app crashes or other issues) and the success of the beacon detection protocol and app notifications across different devices and in different situations. We will collect data on functionality throughout the study, primarily using app-based data and secondarily using qualitative data from interviews with participants and peers at the completion of data collection. *Validity* will be determined by evaluating the concordance between beacon detection and participant EMA reports of peer presence data sources. Details of the project goals and methods for evaluating the goals are described in Table 1.

**Table 1:** Project primary goals and methods for evaluating goals.

Goal			Definition		How determined		Data source
**Functionality**							
	Beacon detection	Whether the peer beacons are detected by the participant app consistent with app settings		Tested in participant orientation to confirm that beacons are detected by participant phone		Beacon Encounter data set App Log data set	
	Beacon encounter notifications	Whether the app functions as expected, defined as delivering report notifications with the expected latency when beacon encounter criteria are met		An indication of notification sent by the server when actual encounters are identified		Beacon Encounter data set Participant Event data set	
	App overall stability	Whether the app functions as expected with minimal errors		App error reports (crash and reinstall) and participant error reports		App Log data set Participant EMA^a^ report data set Postparticipation interview	
	Phone and operating system differences	Whether differences across Android phone and operating system versions are noted		Information about participant phones and operating systems		App Log data set Baseline survey Participant postparticipation survey and interview	
Validity			Concordance between beacon detection and participant report of peer presence		Cross-classification of beacon encounter and participant self-report data		Beacon Encounter data set Participant EMA report data set Participant postparticipation interview Peer weekly surveys

^a^EMA: ecological momentary assessment.

## Methods

### Design

Young adults will participate in a 3-week protocol during which they will complete reports about interactions with peers, including (1) signal-contingent reports triggered by the presence of a Bluetooth beacon being carried by a participant-nominated peer, (2) random reports triggered in time blocks 3 times per day, and (3) a morning report. A baseline assessment will precede field data collection and will aid in identifying peers, and an interview at the end of the study will collect qualitative information and feedback about participant experiences.

### Participants

We will recruit up to 5 participants for our pilot study, and we will recruit 20 participants in the main study, with a conservative estimate of 15 completing the full protocol. The inclusion criteria are as follows: (1) be able to read English, (2) own an Android smartphone with OS11 or newer and have it with them throughout the day (typical for the age group between 18 and 29 years), (3) have a data plan (limited or unlimited), (4) be willing to approach peers to participate, and (5) anticipate not deviating from their typical routine during the study period, including leaving the region (as this would likely reduce exposure to selected peers). Additional inclusion criteria related to our substantive research aims are as follows: (1) aged 18 to 29 years; (2) drinking alcohol with others at least once a week, including drinking >4 (women) and >5 drinks (men) per occasion at least once a week in the past month; and (3) not in or seeking treatment for substance use.

We restricted our project to Android phones because there were several barriers that emerged with iOS. First, iOS places significantly more restrictions on apps that can be put on users’ phones and the methods by which those apps can be loaded onto devices. Relatedly, when provisional software is distributed via the developer side of the App Store, a very limited number of developer testing accounts are provided, limiting the ability to test the app. Second, iOS takes more control than Android over processes that run in the background of the phone, including notifications, which are critical to the functionality of the app. Third, with the availability of the Apple AirTag we were concerned that implementing an alternative Bluetooth Low Energy (BLE) signal detection network with a different beacon would appear in competition with first-party applications and thus our app would be precluded or significantly delayed in distribution via the App Store. Further, as app development for this project started during the COVID-19 pandemic, there was significant focus placed on using BLE for proximity detection by both Apple and Google, with Apple in particular restricting the use of some features. For these reasons, and given the available resources and time frame of the project, we decided early in the course of the project to exclusively develop for Android phones. We note, however, that while owning an Android phone is a requirement for participants, it is not a requirement for peers.

### Procedures

#### Eligibility and Recruitment

Young adults will be recruited from the community through flyers, email listserves, and social media advertisements. A brief web-based screener will establish their initial eligibility. Eligible participants will provide contact information, and a research assistant (RA) will schedule the in-person baseline session.

#### Participant Orientation and Baseline Assessment

A 90-minute in-person session will collect informed consent, demographic characteristics, and alcohol use data and identify possible peer participants. The RA will orient the participant to the project procedures, starting with installing the app on the participant’s phone and recording device characteristics (device manufacturer, model, and Android operating system version). The RA will assist participants in changing settings on their phones to ensure the app will work optimally, including setting Bluetooth, location, nearby devices, and notifications to *on* or *allow*, and checking that settings that pause activity (eg, pause app activity if unused) or remove permissions are *off*. The RA will demonstrate the app interface, including showing the participants how to initiate reports and respond to notifications. The participants will navigate through each report, view examples of response types (radio buttons and text fields), and practice making entries. They will be instructed to respond as soon as possible after a prompt. We will explain that they are expected to keep their phone on, charged, and nearby. We will verify that the participants are not planning on traveling.

#### Peer Eligibility and Recruitment

The inclusion criteria for peers are as follows: (1) at least once a week in a typical week, having meaningful in-person social interaction with the participant. The alcohol-related inclusion criteria for peers are as follows: (2) between the ages of 18 and 29 years, and (3) drink with the participant at least twice a month in a typical month. While not all influential people with regard to alcohol use will be same-age peers, research indicates that same-age peers will most likely be present during drinking events (and in social interactions when drinking might occur) [32-34]. To identify possible peers, the RA will conduct a social network interview (SNI) that involves the participant nominating up to 10 people who they are close to, including friends, family members, or anyone they regularly spend time with in person who is close to their age [35,36]. The participants will report on the characteristics of these individuals, including their age, gender identity, whether they live together, relationship (friend, partner or significant other, casual acquaintance or coworker, sibling or cousin, other family member, and other), and frequency of meaningful social interaction (“How often in a typical month do you spend at least 15 consecutive minutes with this person?”). In line with the substantive study goals, we will also assess the frequency of the network member’s drinking (“How many times in the past month do you think this person drank alcohol?”) and the frequency of drinking with the person (“In the past month, how often did you drink with this person [while both of you were drinking]?”). For this protocol, we will ascertain the participant’s perception of the willingness of the peer to participate in this study.

Working with the RA, the participants will identify 3 peers to be invited to participate in the study based on eligibility. During the baseline session, the participant will attempt to contact each of the 3 selected peers to invite them to participate. If the participant contacts the peer with the RA present and the peer agrees to hear more about the study, the participant will, with the peer’s agreement, share the peer’s contact information with the RA, who will then provide the peer with a web link to the study description and informed consent procedures and will communicate with the peer about their participation from that point forward. If the peer does not respond to the participant in the presence of the RA, the participant will send the peer a brief study description with the web link. If any peer who has received the project description does not respond after 3 days, we will move to the next person on the SNI list and ask the participant to make initial contact with the (new) peer. The participants will be given 5 days to recruit up to 3 peer participants.

**Figure 1:**
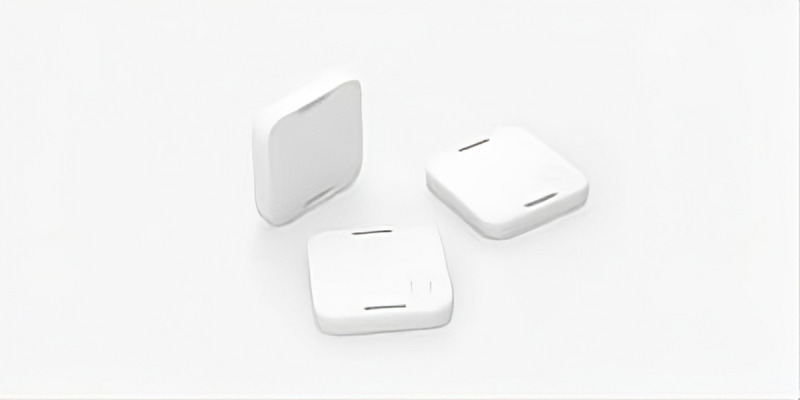
The beacons used in this research were asset tags from Kontakt.io.

Once a peer participant has consented to participate, the RA will arrange a time to give them the beacon and answer any questions. The beacon is 4.4 cm square and 1 cm thick (Figure 1). We will provide a small adhesive patch to allow the peer to attach the beacon to their phone and a ring to facilitate attachment to their keys if they choose. Peers will carry their beacon for 3 weeks of the project. Each beacon is assigned a unique ID that associates the beacon with the participant and peer. The smartphone app reports will begin for the participants once the beacons are distributed to their peers. The peer participants will complete web-based surveys at the end of each of the 3 weeks of data collection, in which they will indicate the days and time of day in the prior week they did not have their Bluetooth beacon with them and times in the prior week they were with the participant. This information will be useful for determining why a beacon was not detected.

#### EMA Reports

##### Overview

Our project will use a custom smartphone app for data collection, developed in conjunction with our developer, MEI Research [37]. The app, which is only available for Android phones, comprises 2 primary components: the participant-facing EMA report delivery component and a background process that continuously scans for the Bluetooth beacons. Researchers create EMA reports and adjust the settings using a web interface. Notification of random and beacon signal-contingent EMA reports is handled locally on-device, so internet connectivity is not needed for either report triggering or beacon detection to function. Content is delivered dynamically from the MEI Research server, which requires cellular or Wi-Fi connectivity. Data are synchronized with the server when the participant opens the app or activates the sync function.

**Figure 2:**
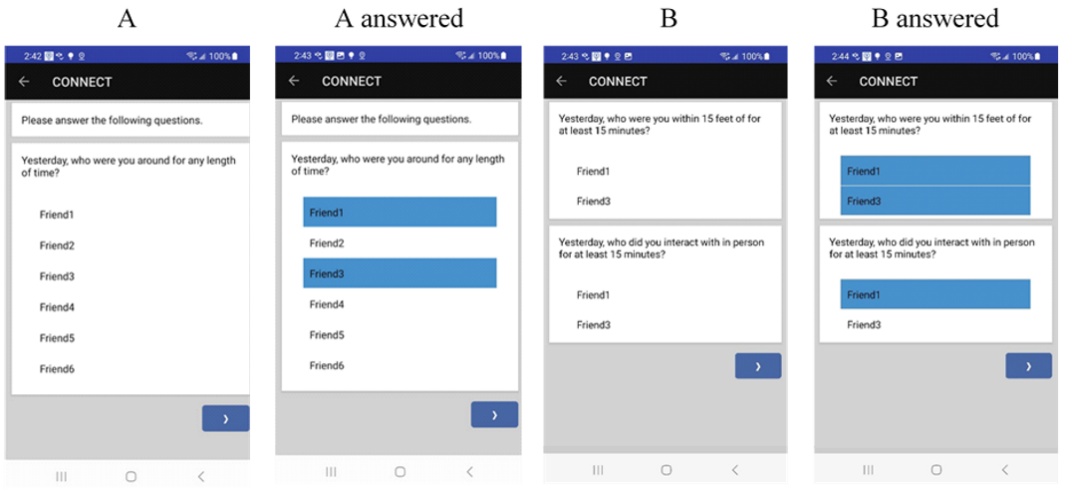
Screenshots of 2 items in the morning survey, first presented without answers (A) followed by the item with friends selected (B). Note that image B presents only friends that were selected in question A.

Before the 21-day EMA phase, the names of the SNI-identified peers and information about their associated beacons are uploaded using the researcher-facing web interface into the app so that the customized list of peers is presented to each participant on EMA reports (refer to the examples in Figure 2). This list of peers will have a maximum of 6 names from the SNI. All peers carrying the beacon will be on the list; others on the list may have declined to carry the beacon or may not have been asked. First, names, nicknames, or initials are presented to maximize confidentiality during data collection. Updating this list can occur in real time without involving the participant, thereby avoiding protocol disruptions. EMA reports begin with an item measuring *peer proximity*: “Yesterday/In the past hour, who were you around for any length of time?” The friend list is presented to the participant for them to choose from, with branching logic used to determine the presentation of subsequent questions. The names or nicknames of peers are not included in the researcher data set; numeric peer IDs (also associated with beacon data) ensure deidentification.

The participants will provide 3 types of EMA reports, completed in 1 to 3 minutes each.

##### Signal-Contingent

*Signal-contingent* reports are triggered by the app according to its detection of beacons carried by peer participants (refer to the subsequent section for details on beacon and trigger settings). We will not have constraints on times when these reports are prompted, and reports will expire (ie, will disappear from the app display) after 1 hour.

##### Random

*Random* reports are intended to sample experiences outside of drinking events and those prompted by signal-contingent reports, including peer contact and influence. The participants will receive 3 random EMA reports per day between 12 PM and 12 AM (12 to 6 PM, 6 to 9 PM, and 9 PM to 12 AM). We chose these intervals to optimize the measurement of alcohol use and exposure to peers. The participants will be informed of these intervals; missing data because of going to sleep is of low concern. Notifications will be followed by reminders at 15 and 30 minutes and will expire after 1 hour. The notification and in-app display for signal-contingent and random reports appear identical on the EMA app interface to minimize unintentional awareness of peer presence. Although there is no basis on which to project a compliance rate with beacon-triggered surveys, we expect a 70% or higher compliance rate with random surveys [31,38].

##### Morning

*Morning* reports will be completed by participants every day. The morning report is always available on the app, with a notification at 10 AM and reminders at 15 and 30 minutes thereafter. The EMA app prioritizes signal-contingent and random reports such that participants are not able to complete morning reports until any pending triggered reports are completed. The morning report items are identical to the signal-contingent and random reports but refer to *yesterday*, whereas the random and signal-contingent reports refer to *in the past hour*. In the morning reports, we also ask about prior-day app functionality related to participant experience (ie, *participant-related functionality*; “Were there times yesterday when you think our system was not working as you expected?”); those who indicate *yes* or *maybe* are asked to describe the issue in a text box. Items also identify possible missing data (eg, “Did you do any of the following yesterday?” with answers “You silenced your phone,” “You turned your phone off,” “You turned off notifications on your phone or for the EMA app,” and “You turned off Bluetooth detection”). During the 3-week EMA period, the study staff will contact the participants once a week to check in, encourage compliance, and address technical issues as needed. The participants can also email, call, or text study staff at any time they have issues or questions.

#### Postparticipation Assessments

After the 3-week data collection period, the participants will complete a modified 15-item System Usability Scale (SUS [39]) with items adapted to our protocol that assess functionality (eg, “The app drained my battery” and “The app worked as expected”) with response options on a 5-point Likert scale from 1=strongly disagree to 5=strongly agree. We will also conduct a semistructured interview to clarify any reported functionality or validity issues. Before the interview, we will examine the participant’s beacon encounter data and reports of peer presence, and during the interview, we will clarify discrepant information, including reconciling signal-contingent reports (ie, indicating beacon detection) at times when the participant did not indicate that the peer was present. We will also probe the reasons for noncompliance and whether triggered report noncompletion appears to be systematically related to peer presence. In this final session, participants will be told how to delete the app from their phone.

### Ethical Considerations

#### Human Subject Protections

The procedures were approved by the Brown University Institutional Review Board (protocol number 2022003448).

#### Informed Consent

The participants and the peer participants will complete informed consent, which includes reviewing detailed consent forms, discussion with the researcher, and documentation of consent.

#### Privacy and Confidentiality

All information obtained during the assessments will be confidential and will be used solely for research purposes. To protect the data and prevent unauthorized access, all EMA data will be encrypted and will remain so until it is accessed by the project staff using a username and password specific to this project. All files with participant-identifying information will be password-protected and stored separately from the data on a server accessed only by the project staff. To preserve confidentiality, we will deidentify data for both peers and participants using a numeric code. The participants will have the researcher provide credentials for the smartphone app that will not include their names, and we will encourage the participants to use phone passwords.

#### Compensation

Participants will be paid US $50 for attending the first session, US $5 per day for answering the EMA reports (at least 2 of 4 of the morning and random reports per day), and US $40 for attending the second session. The participants will also receive a weekly bonus of US $20 if they complete at least 80% of the random and morning reports. The most they can receive for participation is US $255. Peer participants will be compensated US $30 for each week they carry the beacon and answer the weekly survey questions, and US $10 for returning the beacon to the research team, so the most they can receive is US $100. All compensation will be provided in the form of an Amazon e-gift card.

### Beacons and Parameters for Signal-Contingent Triggers

#### Beacon Selection and Features

We prioritized 4 criteria in the selection of the beacon used in this study: signal strength, robustness of the application programming interface, size and convenience of carrying, and battery life. The expected functionality of the beacon is primarily advertising (ie, one-way communication comprising transmission of small packets of data over fixed time intervals for detection and localization by a receiver—here, the EMA app used by the participants) [40]. Given these requirements, we selected BLE, an extension of the traditional Bluetooth protocol. Using BLE facilitated the optimization of our first criterion, signal strength. The Bluetooth protocol includes a feature called Received Signal Strength Indication, an indicator of the signal power received by a device detecting BLE signals. This feature, together with the specifications of the transmission source hardware, enables the computation of approximate positioning in the natural environment, including the estimation of distance [41,42]. Many hardware vendors provide these features; a subset of these vendors provides open, nonproprietary documentation of these values [43] and the ability to interface directly with the beacons through a robust application programming interface, our second criterion. After reviewing options, conducting testing, and consulting with the app developer, we selected Kontakt.io, a company with beacons that met the first 2 criteria and have form factors that can be attached to a phone or keyring to ensure that it is carried consistently. We extensively tested the options available from Kontakt.io and determined that the asset tag was most reliably detected by our app and by generic BLE scanner apps. Its battery life is 6 to 12 months and can be monitored on the internet; therefore, even with reuse, there should be no missing data attributable to a depleted battery.

We initially investigated whether we could build the system such that the Bluetooth on the peers’ phones would be detected by the app on the participant’s phone. For this, an app was needed that could use the BLE functionality within the participant phone to detect and identify the peer phone and prompt the participant to respond within the EMA app. One problem with using the peer phone as the BLE transmitter is that different Bluetooth hardware installed in different phones will report differing RSSI values for the same physical distance since this metric is contingent on the strength of the signal being sent out by the original device. This increases the complexity of the programming logic and would add error into what is a simple threshold check for beacons. Another issue is that we were unable to identify any phone-to-app software with an SDK available to use with our EMA app, whereas beacon detection SDKs were available. There were other considerations, including that in early developmental work concerns were raised by peers about installing an app on their phone, but the most critical were that the resources needed for phone-to-app detection were higher than the beacon-to-app model due to the initial development process, variability of operating systems and hardware, and required ongoing maintenance when relying on operating systems that evolve over time. The key benefits of beacon-to-app detection are the consistency of the technology and lower development costs when using an existing SDK.

#### EMA Report Triggering by Beacon Proximity

**Figure 3:**
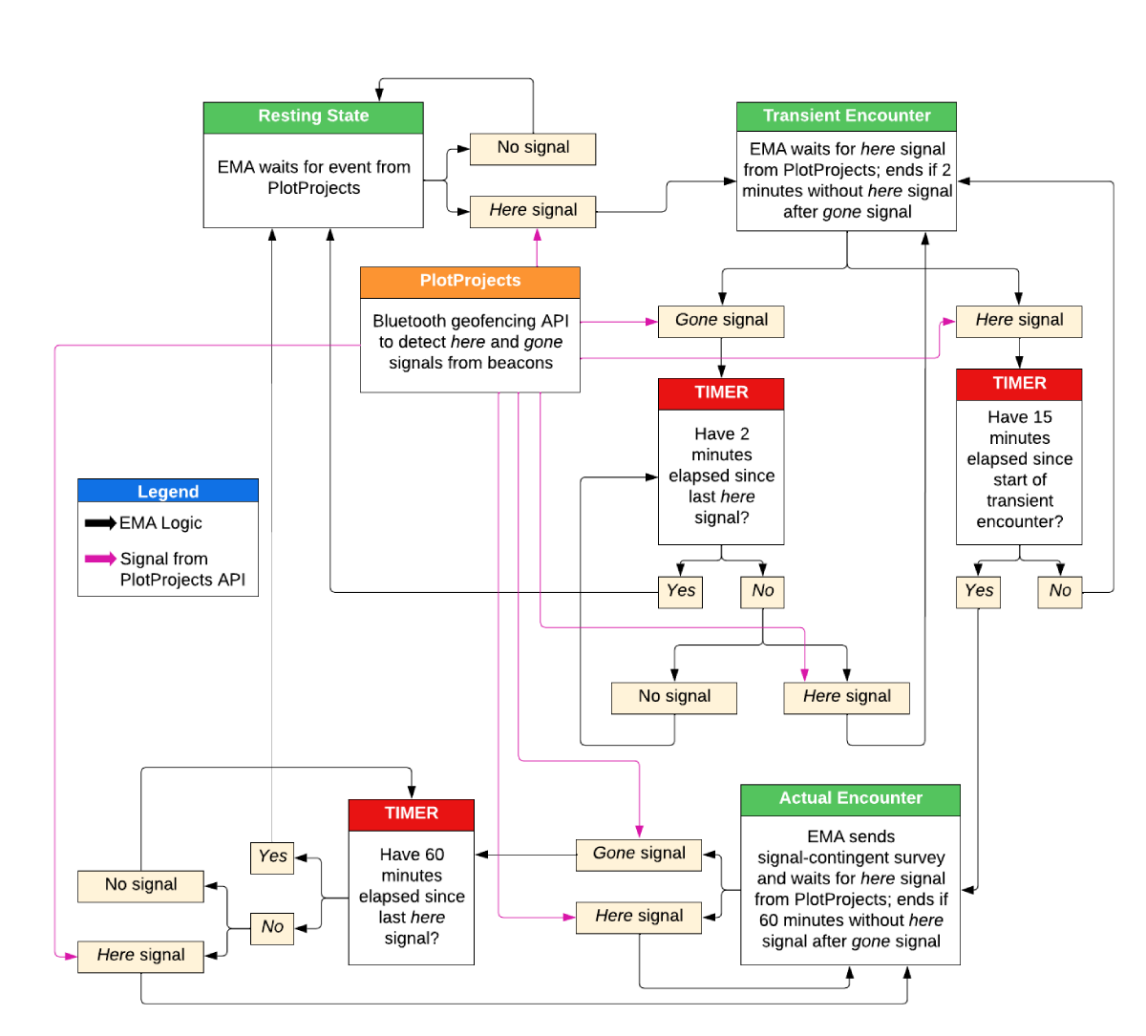
State flow diagram that incorporates the beacon detection, encounter definition, and notifications. PlotProjects is a service and application programming interface used by the app software for the Bluetooth beacon detection.

The protocol for peer proximity detection and subsequent triggering of reports involves the detection of *transient encounters* (ie, incidental, brief social interactions between the participant and a peer identified through signal detection), their conversion into *actual encounters* (ie, meaningful social interactions differentiated from incidental encounters using a time criterion), and their termination as *end encounters* (ie, discontinuation of meaningful social interaction; refer to Figure 3 for a representation of the process). The EMA app continuously scans the participants’ environments for peer beacons. A transient encounter is recorded when the BLE proximity detection service integrated into our EMA app detects a beacon, defined as detecting a signal from a peer beacon that is at least as strong as the Received Signal Strength Indication criteria that correspond to our proximity criterion (ie, 15 ft, 4.6 m). The peer beacon advertising interval, which is the frequency at which beacons send out signals, was set to 1000 milliseconds (1 second) to ensure that the detection corresponds closely to real-world interactions. However, given that the detection of every signal from the peer beacon by the participants’ device during the initial encounter period is not guaranteed, a window of time for the detection of this signal is needed to ensure that detection occurs (ie, that it is not missed, resulting in a false negative); this window was set at 2 minutes for the transient encounter.

A *transient encounter* is converted into an *actual encounter* when the beacon has been detected for at least 15 minutes (our definition of meaningful interaction) without being undetected (ie, gone) for >2 minutes. A signal-contingent report will be triggered upon the conversion of a transient encounter to an actual encounter. Critically, the algorithm that determines triggering by beacon proximity runs separately for each peer participant, such that a transient and actual encounter with a peer beacon can be initiated even when the participant is in an actual encounter with another peer. Given that there will be 3 peers carrying beacons, it is possible that up to 3 reports could be triggered at a time, although we do not anticipate that this will happen frequently. In addition, given our encounter parameters that require 60 minutes of nondetection before another encounter with the same beacon would occur (refer to the subsequent section for explanation), we do not expect the number of signal-contingent reports to be overwhelming but will evaluate this closely.

A separate criterion to end an actual encounter after 60 minutes of nondetection (vs 2 minutes for a transient encounter end) was selected. We selected 60 minutes to reduce spurious nondetection and accommodate behaviors such as going to the restroom or being in different rooms at a party (which may still be part of an ongoing meaningful interaction despite not meeting the proximity criterion). Although the duration of the time criteria (ie, 15 minutes for the actual encounter and 60 minutes for the end encounter) were somewhat arbitrary, the evaluation of the sensitivity and specificity of the criteria via concordance with self-report is a goal of the study. Subsequent work can trial different definitions based on study findings, as well as what contact is truly influential and whether the length of influential contact varies by relationship type. Table 2 presents our initial criteria (far-right column) based on the best judgment, but the criteria are modifiable within the researcher-accessed interface.

**Table 2:** Transient and actual beacon encounter terms, definitions, and criteria.

Term	Definition	Criteria
Encounter	Incidental or purposeful social interaction between the participant and a peer is identified through BLE^a^ signal detection with a predefined proximity criterion.	15 ft (4.6 m)
Start of a transient encounter	Brief social interaction. A transient encounter begins but does not constitute meaningful interaction until it converts to an actual encounter.	1000 milliseconds
End of the transient encounter	End of a brief social interaction is defined by a given time criterion. Intended to ensure that very brief encounters do not prompt a notification.	2 minutes without beacon contact
Start of an actual encounter	Meaningful social interaction defined by a given criteria of time. A transient encounter is converted to an actual encounter. An actual encounter triggers a signal-contingent report.	15 minutes of beacon contact with no periods >2 minutes without beacon contact
End of the actual encounter	Discontinuation of meaningful social interaction.	60 minutes without beacon contact

^a^BLE: Bluetooth Low Energy.

There are several technology-related considerations for the detection of meaningful interactions. First, an a priori definition of a meaningful social interaction had to balance sensitivity for detecting such events (addressing the *feasibility* of detecting social interactions) with specificity in excluding incidental, nonmeaningful interactions (and reducing the burden of responding to multiple signal-contingent reports). However, our a priori criterion of requiring 15 minutes for a transient encounter to be converted into an actual encounter will miss brief but meaningful interactions (eg, consuming a shot of hard alcohol together, offering alcohol). Importantly, we designed our protocol to minimize missing data associated with such events by including the assessment of past-hour peer presence in our random reports and past-day peer presence in our morning reports. This design will allow us to cross-tabulate the occurrence of self-reported interactions of even short duration (as recorded in the EMA report data set) with the occurrence of all beacon detections, including transient encounters (as recorded in the Beacon Encounter data set).

### Data Sets and Calculated Variables

#### Overview

Data produced by the app, including information about encounter events, participant self-reported data, and metadata, are stored in several data sets. These data sets are supplemented by postparticipation self-report data from (non-EMA) surveys and semistructured interviews. Table 3 contains a description of the data sources; Table 1 links the data sources with the study goals and methods to the evaluate goals.

**Table 3:** Metadata and calculated variables and associated source data sets that will be used to evaluate research goals.

Metadata or calculated variable	Source data set					
	App log	Beacon Encounter	Participant events	Notification log^a^	EMA^b^ report	Postparticipation
Participant ID	✓	✓	✓	✓	✓	✓
Peer ID (corresponds to beacon ID)		✓			✓	✓
Event or Report timestamp	✓	✓	✓	✓	✓	✓
Event type (eg, action delivered, participant report started, report response, and app synced)^c^	✓	✓	✓		✓	
Session ID (helps link together events from different data sets)		✓	✓	✓	✓	
Source of event (eg, morning or random report)			✓	✓	✓	
Corroborative self-report					✓	✓

^a^Contains only notifications for morning and random reports.

^b^EMA: ecological momentary assessment.

^c^Metadata recorded by the app and stored.

Further information about the source data sets are presented in the Multimedia Appendices 1-5.

#### App Log

This data set contains information about the app version used and (morning, random, beacon-triggered) report items that are programmed into the app. It also contains event types (eg, app opened and app synced) and errors (eg, when the operating system denies permission to the app). We will use information from this data set to determine that the app was installed correctly and that it was functioning as expected during the study.

#### Beacon Encounters

Variables include participant ID, beacon ID, start and end timestamps of transient and actual encounters, the timestamp when the transient encounter was converted into an actual encounter, and the timestamp for when the report notification was sent. A unique session ID links the elements (start and end) of given (transient and actual) encounters.

#### Participant Events

Variables include participant ID and types of events (eg, report start, report submit, and report notification) with timestamps. The report start and submit timestamps are present for all reports, including random, morning, and signal-contingent reports. Report notification timestamps (1) duplicate the timestamp of the transient-to-actual conversion reported in the Beacon Encounter data set (which triggers a notification) and (2) indicate whether other notifications are for random or morning reports.

#### Notification Log

Variables include session ID, report type (ie, random and morning), and timestamps when timed notifications and reminders were scheduled to be sent.

#### EMA Reports

Data include participant responses to items in the signal-contingent, random, and morning reports, including indicators of who among their peers is or was present and self-reports about app functionality. For example, in each report, we ask, “Who did you interact with in person for at least 15 minutes?” The report type and timestamp will be used to ensure that data responses are aligned with information from other data sets and reports.

#### Postparticipation Data

Data from the postparticipation interviews and the SUS will be used to evaluate the app’s functionality. Participant interviews will also be used to investigate identified discrepancies between the beacon-derived data and participant reports (ie, validity) and will be supplemented with weekly survey day-level reports from peers about their proximity to participants.

### Data Analysis Plan

#### Functionality of Beacon Detection by the Participant’s Smartphone

We will confirm the detection of the 3 beacons by the participant’s phone during the orientation. We expect to observe smartphone notifications for each beacon when the transient encounter is converted into an actual encounter (after 15 minutes). If notifications are not observed, we will examine the Beacon Encounter data set to determine whether transient encounters are being recorded (ie, whether the beacon is detected by the app) without being converted into an actual encounter. Functionality will be evaluated with 3 metrics: (1) the proportion of participants whose phones detect all beacons during the orientation, (2) the proportion of beacons detected by participant phones, and (3) the latency between exposing the participant phones to the beacons and the start of a transient and actual encounter. These observations will be systematically recorded and aggregated across beacons and participants.

#### Functionality of the Beacon Encounter Notifications

Data from the Beacon Encounter data set will be used to evaluate functionality in two ways: (1) the proportion of actual encounters for which a signal-contingent notification was shown as sent by the server in the Beacon Encounter data set, and (2) the mean latency in minutes between the conversion to an actual encounter and the sending of the signal-contingent notification. The functionality of notifications for random and morning reports will not be evaluated, as these are commonly used in EMA and are fully functional in the app.

#### Functionality Related to App Stability

The app log, the EMA data, and the end-of-study interview and survey will be used to assess the participant-experienced functionality. The app log contains details about the events that have occurred within the app, including information about app crashes and app reinstalls. Using data provided by participants in the morning report, participant-related functionality will be determined as follows: (1) the total count (ie, person-days) of participant-reported suspected functionality problems, (2) the person-level proportion of days of suspected functionality problems, and (3) aggregated item-level and overall mean scores on the adapted SUS. Open-ended feedback will also be obtained in the morning report as well as in the end-of-study interviews.

#### Functionality Related to the Phone and Operating System

The manufacturer, model, and Android operating system of the participant devices will be examined as moderators of the above metrics of functionality. The variables will be evaluated using a series of Mann-Whitney *U* tests (for means and counts) and chi-square tests (for comparing proportions) for each metric. Considering the small sample size, the groups will be driven by the sample of the device itself. For instance, the manufacturer could be binned to *Samsung* (or potentially *Google*) versus *all other manufacturers*; the model could be binned to *currently supported* (ie, receiving ongoing feature and compatibility updates) versus *legacy*; and the operating system could be binned to *Android 13* (the newest) versus *Android 11 or 12* (operating systems that were used during app development). Independent of group comparisons, descriptive findings on functionality will be aggregated for each manufacturer and operating system version.

#### Validity

We define validity as the concordance between encounters recorded in the Beacon Encounter data set and participant reports of peer presence recorded in the EMA reports (Table 4).

**Table 4:** Beacon validity: cross-classification of beacon encounter and participant self-report data^a^.

			Participant self-report data		
			Positive (participant reports peer within 15 ft, 4.6 m for 15 minutes)		Negative (participant does not report peer nearby)
**Beacon detection**					
	Positive (signal-contingent trigger)	True positive: beacon detected and triggers signal-contingent report and participant reports peer presence in signal-contingent report (*sensitivity*)		False positive^b^: beacon detected and triggers signal-contingent report but participant does not report peer presence in signal-contingent report (*specificity; protocol criterion validation*). This could happen if: delay or error in notification (*technology failure*) peer is not actually present (*technology failure*) peer is present with beacon but the index participant is not aware (*inaccurate index participant self-report*) peer is present with beacon but not when the participant answers report (*limitation of detection settings*)	
	Negative (no signal-contingent trigger)	False negative^c^: beacon never detected but participant reports peer presence on next EMA^d^ random report (*sensitivity*). This could happen if: peer is present with beacon but beacon is not detected (*technology failure*) peer is present without beacon (*peer noncompliance*) inaccurate index participant self-report peer beacon is detected in transient encounter but criteria for actual encounter are not met so does not trigger signal-contingent report but participant reports peer presence on EMA random report (*specificity; protocol criterion validation*)		True negative: beacon never detected and no report of peer presence on EMA random report (*sensitivity*) peer beacon is detected in transient encounter but does not trigger signal-contingent report (criteria for actual encounter are not met) and no report of peer presence on EMA random report or morning report (*specificity; protocol criterion validation*)	

^a^Concordance will be evaluated only when self-report EMA data are available. Therefore, beacon data collected when index participants are not compliant with random or signal-contingent reports will not be usable in the evaluation of concordance, as no self-report of peer proximity will be available.

^b^Reasons for false-positive signal-contingent triggers may not be known to the study team.

^c^Reasons for false-negative signal-contingent triggers may not be known to the study team.

^d^EMA: ecological momentary assessment.

*False negative* beacon detections will be evaluated in a series of steps: First, we will compute the proportion of (1) days and (2) random reports in which a participant reported peer presence (from the EMA data set), but a corresponding transient or actual encounter does not exist (from the Beacon Encounter data set). If a transient encounter, but not an actual encounter, is recorded, we will compute (3) the proportion of these false negative events that occurred because of the a priori selection of 15 minutes as the threshold delineating a transient versus an actual encounter (based on timestamps of encounter events in the Beacon Encounter data set). For the false negative events that are not associated with a transient encounter, we will further attempt to delineate the reason underlying this false negative as either technology-related (the peer was carrying the beacon but the beacon was not detected) or compliance-related (the peer was not carrying the beacon) by integrating self-report data on compliance from the peer weekly surveys. If sample size and base rates allow, we will also fit a series of generalized linear mixed models [44] regressing concordance at the person level (eg, sex and age) and proximal prior event level (eg, context and alcohol use) predictors to identify systematic factors associated with detection failure. *False positive* beacon detections, as indicated by the triggering of a signal-contingent report when the peer beacon is not present, have never occurred in preliminary testing. Given the factors needed to incur a false positive detection, which should only occur if beacon information was entered incorrectly by the research team, we believe that such events are highly unlikely. However, it is possible that a notification could be delayed by the operating system or other action (eg, if the index participant phone is off), which would result in the participant report being delayed, and thus the participant report would not confirm the peer presence. Beacon detection also could occur when a peer was present but the participant was not aware (eg, at a large party) or if the peer left the area before the participant answered the report. We will compute the proportion of signal-contingent reports in which participants deny a past-hour peer presence. We expect that the participant interviews and the peer surveys will be informative about the conditions under which false positives occur and will improve our understanding and measurement of peer proximity and influence. Ultimately, the detection of an encounter by the protocol when such an interaction did not occur from the participant’s perspective provides critical information necessary for the computation of the specificity and positive predictive value of our detection protocol.

#### Anticipated Sources of Missing Data

With various momentary assessment reports and passive data collection from multiple peers, the source of missing data is important to identify. We expect to have missing data owing to technical issues for the following reasons: (1) the beacons could stop sending a signal because of low battery or hardware failure; (2) the app could crash or be unable to send or receive data; (3) the phone operating system could update, resulting in the app or phone features not working as expected; or (4) the software detection algorithm may stop identifying encounter start and end signals. Missing data may also be because of participant or peer noncompliance, including the following: (1) participant nonresponse to EMA reports, (2) participant changing phone settings or status such that the detection of beacon signals or notifications is interrupted, or (3) peer failure to carry the beacon. Items in the morning report, postparticipation surveys and interviews, and the peer weekly survey will capture times when the participant or peer is aware of these issues occurring. While imperfect, this information should allow us to characterize situations in which the reason for missingness is known and thus maintain the missing-at-random assumption. Situations in which we are not confident about the reason for missing data will be investigated for systematicity using proximal prior predictors in the EMA data set to further maintain the missing-at-random assumption.

## Results

Participant enrollment began in February, 2023. As of submission of the manuscript (June 2023), nine participants and 21 peers were enrolled, with data collection anticipated to finish in December, 2023. Data analysis will begin immediately upon completion of data collection.

## Discussion

### Principal Findings

The research described in this paper will advance the literature by developing and validating a passive detection system for situations in which social influence is important. While this Bluetooth-based technology could be applicable to many research, clinical, or community settings in which social contact is relevant, our substantive goal is to facilitate research characterizing the real-time social context of alcohol use and, in turn, to inform the timing and context of mobile-delivered interventions to reduce hazardous drinking [45,46]. In this paper, we describe our planned procedures for evaluating the functionality and validity of the system, which incorporates multiple components of functionality and uses data from the app and reports from participants and up to 3 of their close peers. Our approach to collecting user-reported information along with passively recorded data is consistent with recommendations for identifying high-risk events and the timing of real-time intervention [47].

Just-in-time adaptive interventions target behavior in the natural environment at a time when behavior is opportune for modification [48]. These tailored mobile-delivered interventions provide the right type and timing of intervention in response to real-time behavior, cognition, or context to prevent negative health outcomes [48-50]. Ideally, interventions could incorporate this BLE-based peer proximity approach to help people who are interested in changing behaviors by identifying contexts that confer greater risk (or alternatively, that protect against risk), alerting these people or others about the risk inherent in their present situation, and including encouragement and information about how to avoid the situation. This approach could be combined with other sensor technology, including location detection or geofencing using GPS to detect proximity to risky locations (eg, alcohol outlets), and other smartphone features (eg, the smartphone accelerometer to measure movement or engagement with messaging and texting apps) [27]. This study will provide much-needed information about the appropriate timing and context for just-in-time adaptive interventions in a scenario that is highly characteristic of young adult drinkers: consuming alcohol with close peers.

### Limitations

Limitations related to technology include the need to build the system with a contracted app developer; to the best of our knowledge, there are no systems that researchers could use out of the box for person-proximity detection and EMA report triggering. The system only works with phones that use the Android operating system; iOS imposes barriers to the development of technologies and the distribution of test builds, whereas Android allows for the installation of third-party software outside of the context of Google Services (ie, sideloading). However, Android had multiple operating system updates while our project was under development; each update required a revision of the app and additional testing. This is an unavoidable element of app development that adds considerable time and resources to a project. We considered providing Android phones to participants who did not have them but had concerns that intermittent or limited use of a secondary phone would reduce both compliance and app functionality (eg, fewer notifications being sent to phones frequently in sleep mode). Another technology limitation is our inability to implement a minimum time interval between random and signal-contingent reports or to set a limit on the number of signal-contingent reports a participant receives. We will evaluate the average number of reports and intervals between reports and hope to include adjustments in future iterations.

Our design will not detect influential peers who are not carrying a beacon; therefore, its success will rely largely on how well our SNI identifies influential peers and whether they participate. Our procedures will also poorly detect friends who are influential through interactions other than in-person contact (eg, via social media or text). Influence could also occur more quickly than the time duration estimate that we defined in the protocol; this influential interaction would be missed. During all EMA reports, we collect information about peers who are present but not carrying a beacon (identified a priori) and peers who are present but not on the participant’s selected peer list, which will help us to determine how successful our SNI selection process was and address some of the above limitations; we also expect that information collected in the postparticipation interviews will assist us in adjusting procedures for identifying influential peers.

We are relying on self-report to a considerable extent in that we are using it as the ground truth for whether a beacon detection is accurate or not. Self-report can be inaccurate or missing, but it is the standard in the field for describing context and alcohol use, and there are no alternatives that we could use to compare to beacon detection. While we are confident that our informed consent procedure, the provision of a clear rationale to participants and peers, and confidentiality protections will minimize inaccurate reporting, it is possible that social desirability or other person-level or contextual effects in some situations will result in missing or inaccurate reports of peer presence or alcohol use [51,52]. We expect that we will not be able to identify the reason for some disagreements between beacon detection and self-report, but the very robust data sets will allow for sensitivity analyses that will help us identify and understand discrepancies. For example, with morning reports and sensor data, we can compare the concordance between beacon detection and self-report on drinking versus nondrinking evenings. The weekly web-based surveying of peers was designed to minimize burden (and thus increase the likelihood of peer inclusion), but may produce unreliable data about the time spent with the participant.

The success of this research relies on participant and peer compliance, as their reports are needed to evaluate every project goal. Although some degree of noncompliance with EMA is common [38], poor compliance would limit our ability to evaluate functionality (refer to Table 4 for details) independent of systematic bias owing to missingness in the data collected. Although our participant burden is consistent with the current practice in EMA research, our peers must consistently carry a small beacon. Our requirement that peers continuously carry a beacon will likely result in more missing data than if there were an app for peers to install on their phone (which would also be easier to do remotely). Finally, this type of work requires considerable staff time for participant and peer recruitment (up to 3 peers per participant), monitoring incoming EMA and beacon data, and data management and analysis.

### Future Work

In addition to the evaluation described herein of functionality and validity, we are collecting participant feedback about our procedures, which will allow us to evaluate feasibility and acceptability. Together, this information will inform a large-scale study with a representative sample, a wider age span, and a broad representation of social context, permitting the study of dynamic intersections of multiple determinants of drinking behavior, including social context, location context, and individual factors (eg, craving, affect, and motivation) that underlie contextual influences [53].

## Appendix

Multimedia Appendix 1 Application (App) log data set.

Multimedia Appendix 2 Beacon encounter data set.

Multimedia Appendix 3 Participant events data set.

Multimedia Appendix 4 Notification log data set.

Multimedia Appendix 5 Ecological momentary assessment report data set.

## Abbreviations

BLE: Bluetooth Low Energy
EMA: ecological momentary assessment
RA: research assistant
SNI: social network interview
SUS: System Usability Scale

## Data Availability

Data sharing is not applicable to this paper as no data sets were generated or analyzed in this protocol. Once data are generated, data sets will be available from the corresponding author on reasonable request.
