# Supplementary figures and images for: A Bluetooth-Based Smartphone App for Detecting Peer Proximity: Protocol for Evaluating Functionality and Validity
Source: JMIR Res Protoc. 2024 Apr 5;13:e50241. doi: 10.2196/50241 (PMC11031693; doi:10.2196/50241)

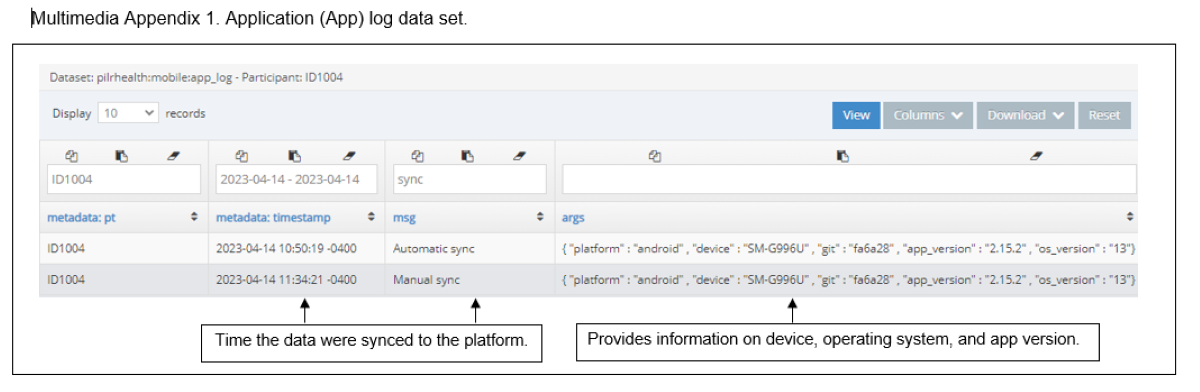

Supplement: Multimedia Appendix 1 [file resprot_v13i1e50241_app1.png]

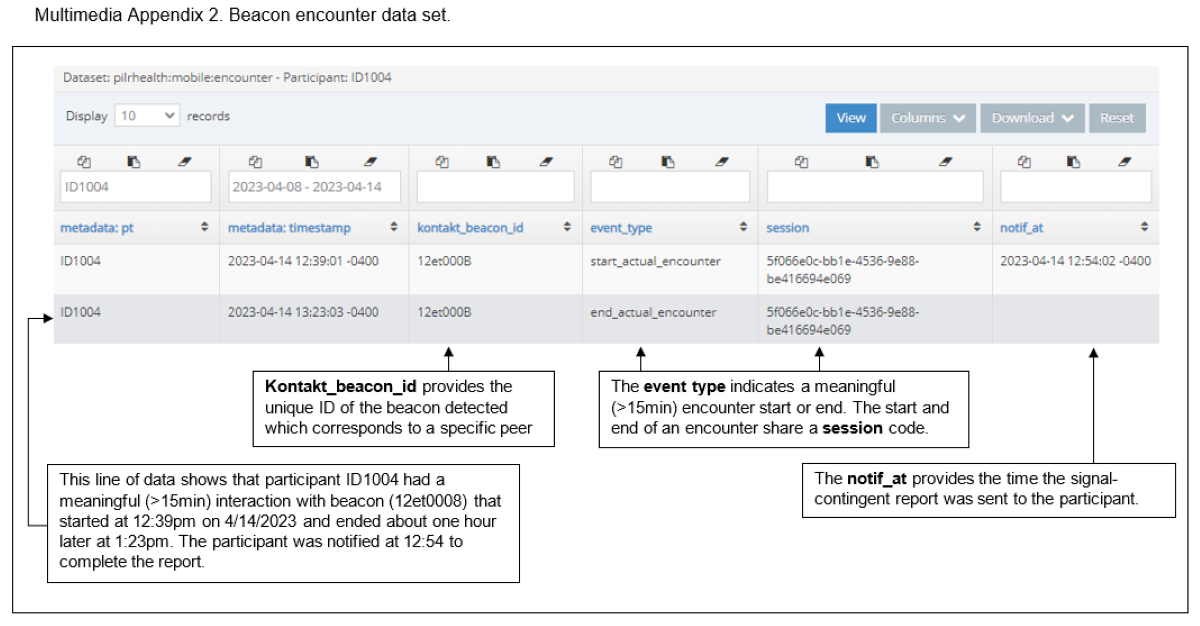

Supplement: Multimedia Appendix 2 [file resprot_v13i1e50241_app2.png]

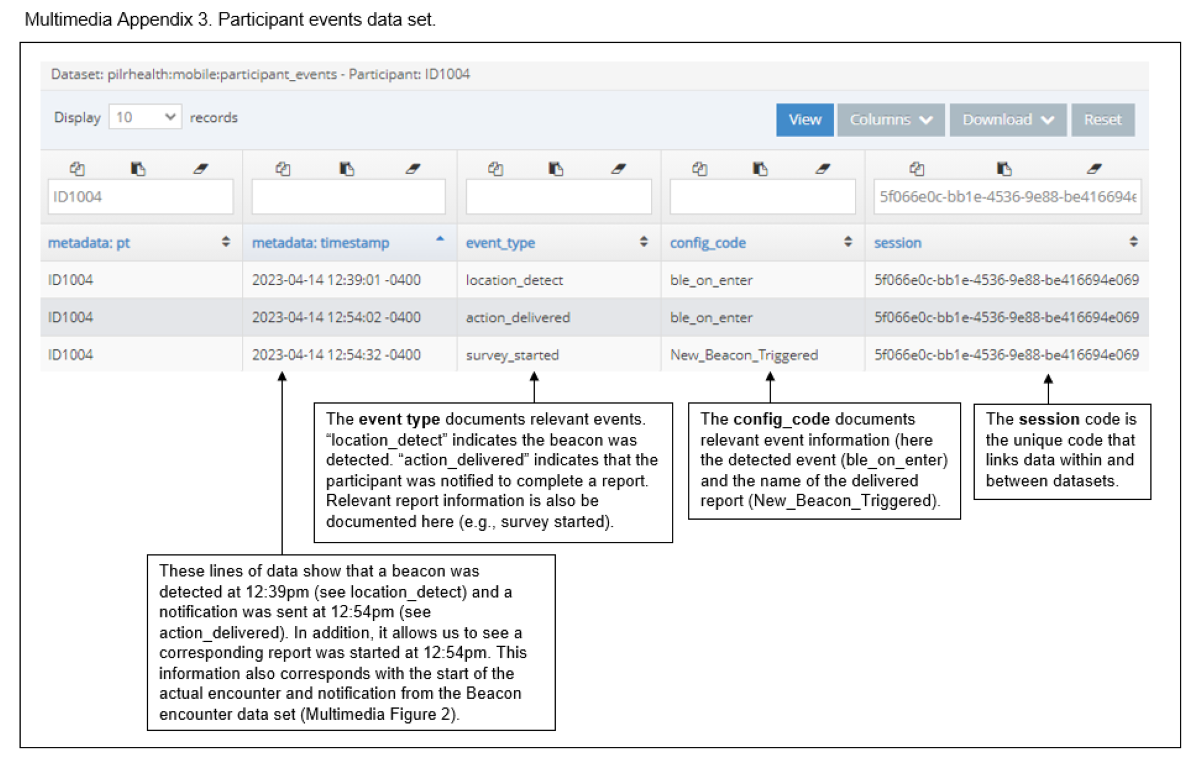

Supplement: Multimedia Appendix 3 [file resprot_v13i1e50241_app3.png]

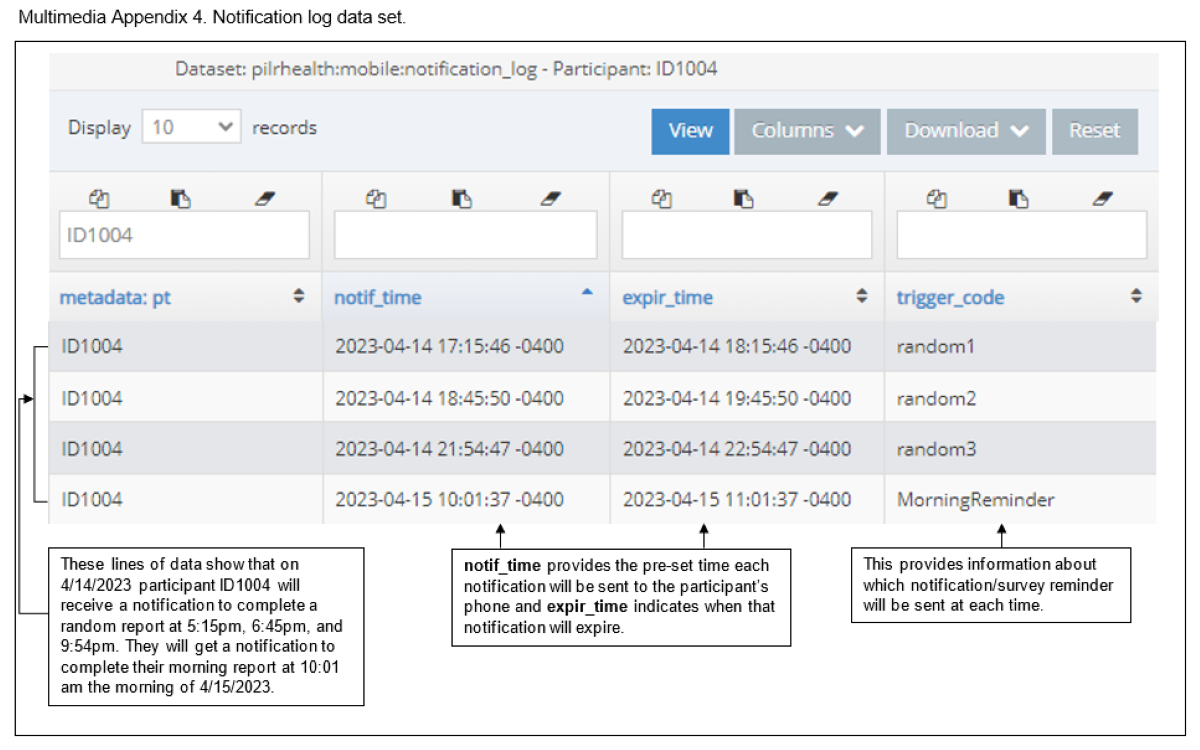

Supplement: Multimedia Appendix 4 [file resprot_v13i1e50241_app4.png]

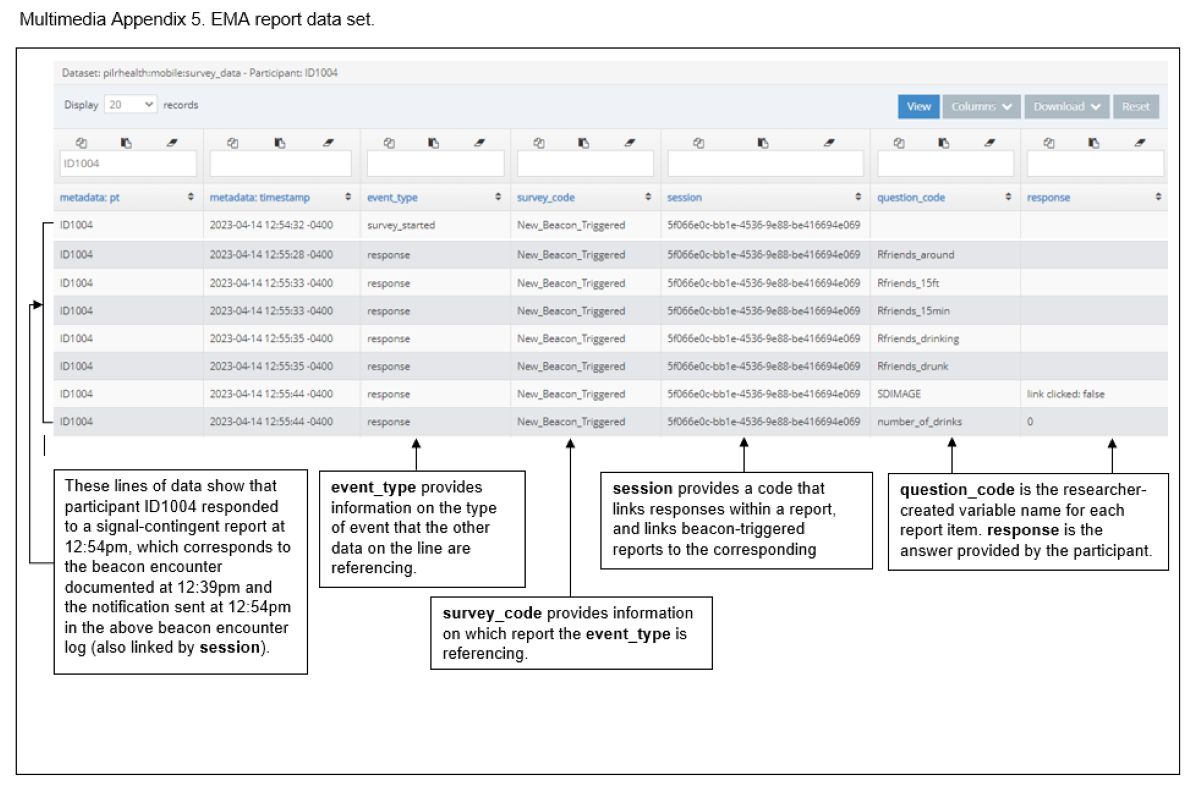

Supplement: Multimedia Appendix 5 [file resprot_v13i1e50241_app5.png]
